# Intranasal delivery of iron chelators and management of central nervous system disease

**DOI:** 10.3389/fphar.2025.1709259

**Published:** 2025-12-18

**Authors:** Ruiying Cheng, Jonghan Kim

**Affiliations:** Department of Biomedical and Nutritional Sciences, University of Massachusetts Lowell, Lowell, MA, United States

**Keywords:** iron chelation, intranasal delivery, neurodegenerative diseases, blood-brain barrier, brain delivery

## Abstract

Brain iron dyshomeostasis plays a critical role in the pathology of multiple central nervous system (CNS) disorders, including neurodegenerative and neuropsychiatric diseases. Iron chelators such as deferoxamine (DFO) and deferiprone (DFP) have demonstrated therapeutic potential in mitigating disease progression in these conditions. However, systemic administration is hindered by poor blood-brain barrier (BBB) permeability, dose-limiting toxicity, and poor patient compliance due to frequent dosing regimens. In recent years, intranasal (IN) drug delivery has emerged as a promising strategy to bypass the BBB, providing a direct nose-to-brain delivery route via olfactory and trigeminal pathways while minimizing systemic exposure. This review provides a comprehensive summary of the current status of iron chelation therapy for CNS disorders with a focus on pharmacokinetics, efficacy, and translational potential of IN administration. While IN DFO has been extensively studied in preclinical models of Alzheimer’s disease and stroke, recent developments have expanded the scope to other chelators such as DFP. We compare traditional systemic routes, including oral and intravenous, with intranasal administration, highlighting their respective advantages and limitations for CNS delivery. With ongoing advances in formulation and delivery technologies, IN iron chelators provide a promising alternative for the treatment of CNS disorders characterized by impaired iron homeostasis in the brain.

## Graphical Abstract

**Figure:**
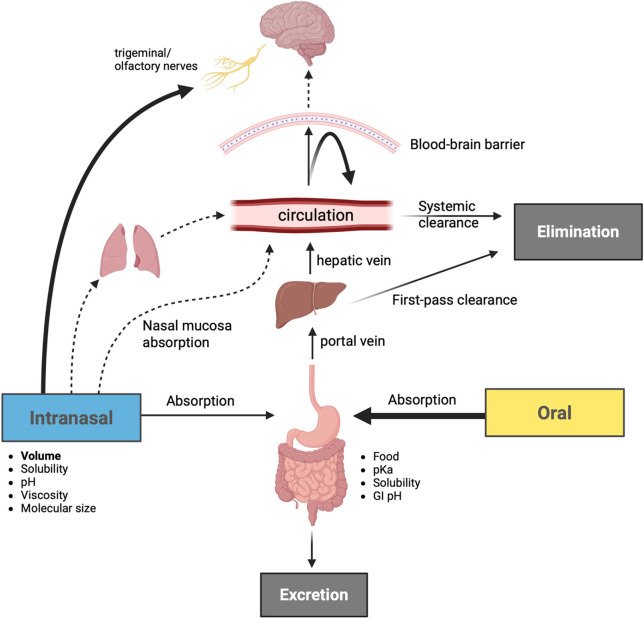

## 1 Introduction

Neurological conditions, including central nervous system (CNS) disorders, have emerged as the leading cause of illness and disability worldwide, affecting more than 3 billion individuals – more than one-third of the global population – based on a study in 2021 (GBD, 2021 Nervous System Disorders Collaborators, 2024). The cases of brain disorders by 2050 are projected to have a 22% increase from 2021 estimates, with the most prevalent conditions being migraine, Alzheimer’s disease, depressive and anxiety disorders, and strokes (Lei and Gillespie, 2024).

Disrupted iron homeostasis in the brain has been increasingly implicated in the pathology of many CNS diseases, where excessive iron accumulation can lead to oxidative stress, neuroinflammation, and ferroptotic cell death in vulnerable brain regions (Salvador et al., 2010). Therefore, iron chelators have been investigated as potential therapeutic agents to mitigate the iron-related neurotoxic conditions.

Effective treatments for neurological disorders are still lacking, primarily due to the presence of the blood-brain barrier (BBB), a unique and selective endothelial barrier that protects against neurotoxic substances, as well as prevents the movement of xenobiotics into the CNS, including pharmacological agents (Crowe et al., 2018). Consequently, the BBB allows only small, lipophilic molecules or those with specific transporters to pass through. As a result, it blocks 98% of small molecules and nearly 100% of large molecules from entering the brain (Banks, 2009; Pardridge, 2005). Iron chelators, such as deferoxamine (DFO) and deferiprone (DFP), have shown potential in reducing iron-induced oxidative stress and modulating iron levels (Guo et al., 2019; Zhu et al., 2022; Wang C. et al., 2023; Dusek et al., 2016). However, traditional systemic drug delivery to the brain is limited by the BBB, requiring higher dosage or prolonged treatment schedules that can lead to suboptimal brain concentrations and potential systemic side effects. The limited success of drugs used in neurological disorders has led to the exploration of optimized drug delivery systems to overcome this barrier (Cecchelli et al., 2007).

Approaches such as intrathecal, intracerebroventricular, and intraparenchymal injections, as well as transient BBB disruption, have been employed to deliver drugs with limited CNS bioavailability (Ganger and Schindowski, 2018; Rezaee et al., 2025). While these invasive techniques provide direct access to the brain, their clinical use is limited by the complexity of dosing and poor feasibility in outpatient settings. As a result, recent strategies have been shifted toward developing non-invasive drug delivery methods to improve brain drug delivery, including the use of prodrugs, nanoparticles, focused ultrasound, and intranasal (IN) delivery methods (Terstappen et al., 2021). Among these, the IN route has gained increasing attention as a promising tool to bypass the BBB and deliver therapeutics (including iron chelators) directly to the CNS (Crowe and Hsu, 2022). The most recent review on intranasal chelation was published in 2021 by Farr and Xiong, summarizing a decade of preclinical and translational studies on intranasal DFO in neurodegenerative and cerebrovascular conditions (Kosyakov et al., 2021; Farr and Xiong, 2021). However, since then, the field has continued to progress rapidly, with recent animal studies and early-phase clinical trials that highlight the efficacy and feasibility of IN delivery for iron chelators (Boyuklieva et al., 2024; Amoushahi et al., 2024). In the meantime, the broader field of IN drug delivery has witnessed exponential growth with frequent publications about other novel IN formulations and delivery technologies for CNS-targeted therapies. Therefore, this review paper discusses the current state of iron chelation therapy on CNS conditions with a focus on the potential of intranasal delivery. We will explore its possibilities and advantages, as well as limitations and toxicological considerations, providing a comprehensive overview of this promising method of brain-targeted drug delivery.

## 2 Impaired iron homeostasis and brain dysfunction

Iron is essential for multiple biological functions, including oxygen transport, redox actions, and DNA synthesis (Kühn, 2015). Iron is also essential in the CNS, required for neuronal myelination, neurotransmitter synthesis, and electron transfer, playing a vital role in brain development and function (Beard et al., 2009). For example, iron is a cofactor for enzymes involved in the synthesis and signaling of neurotransmitters, including dopamine and serotonin (Hare et al., 2013). Under normal conditions, iron status is tightly regulated in the brain, primarily via transferrin receptor-mediated transport and ferroportin-mediated export (Rouault and Cooperman, 2006). This homeostasis ensures that sufficient iron is available for vital processes, while abnormally high or low iron levels can disrupt neuronal function directly or indirectly linked to several neurodevelopmental and neurodegenerative diseases (Levi et al., 2024; Wu et al., 2023).

### 2.1 Iron deficiency in the brain

Iron deficiency is the most common micronutrient deficiency worldwide, common in pregnant women and young children due to their high iron demands (Stevens et al., 2013). In the brain, iron deficiency can occur without significant changes in iron levels in peripheral tissues, leading to imbalanced neurotransmitter homeostasis, decreased myelin production, impaired synaptogenesis, and declined function of the basal ganglia (Pivina et al., 2019). Cognitive functions in learning and memory are also compromised due to iron’s critical role in hippocampal development and myelin sheath formation. Iron deficiency is linked to early neurological and psychiatric conditions such as autism spectrum disorder (ASD), attention-deficit/hyperactivity disorder (ADHD), and major depressive disorder (MDD) (Wiegersma et al., 2019; Yang et al., 2021; Mills et al., 2017; Leung and Kyung, 2024). Treatments include dietary interventions to enhance iron intake from iron-rich foods and oral iron supplementation, despite potential gastrointestinal side effects (Gisslen et al., 2023).

### 2.2 Iron overload in the brain

In contrast to iron deficiency, excess iron in the brain is neurotoxic due to its ability to produce reactive oxygen species (ROS) and resultant oxidative stress (Salvador et al., 2010). Iron overload in the brain poses significant risks in neurological conditions, including neurodegenerative diseases such as PD and AD (Zecca et al., 2005; Quintana et al., 2006). Disruptions in iron homeostasis in the brain can also be a risk factor or exacerbate neuropsychiatric conditions, including depression, anxiety, and cognitive impairments (Al-Hakeim et al., 2020; Lotan et al., 2023; Chen et al., 2023), potentially due to iron’s ability to modulate levels of neurotransmitters both directly and indirectly (Kim and Wessling-Resnick, 2014). While the role of iron deficiency is well-characterized in neuropsychiatric conditions, emerging evidence also suggests that iron overload may contribute to certain mood and behavioral abnormalities (Wu et al., 2023). An early clinical report in 1994 described significant clinical improvements in patients treated with DFO (Cutler, 1994). Later studies have explored potential links between brain iron overload, ferroptosis, and mood disorders, as reviewed by Duarte-Silva et al. (Duarte-Silva et al., 2025). Iron overload has also been observed in rare cases of children with inborn brain neurodegeneration due to iron accumulation (NBIA) associated with autism-like conditions (Zigman et al., 2021) and anxiety-like behaviors (Cutler, 1994; Maaroufi et al., 2009), although the causal relationship remains obscure. Despite the delicate regulation of iron import, storage, and export in different brain cell types, the brain lacks a robust mechanism for iron excretion. As a result, passive iron removal by iron chelators is crucial for normalizing excess brain iron. In many neurological disorders, abnormal iron accumulation is one of the common features, although it remains debated whether iron is a driving cause or a consequence of the disease process (Levi et al., 2024).

### 2.3 Molecular mechanisms of iron-associated brain disorders

The underlying mechanisms linking aging, neurodegeneration, and iron accumulation are not yet fully understood. Excess iron catalyzes the aggregation and hyperphosphorylation of β-amyloid and tau proteins, which are the major pathologies of AD, contributing to senile plaques and neurofibrillary tangles (Levi et al., 2024). Elevated iron levels are also linked to oxidative stress and ferroptosis, a programmed cell death dependent on intracellular ferrous iron and impaired antioxidant defense systems (Abdukarimov et al., 2025). This process can be further exacerbated by neuroinflammatory processes, including chronically activated microglia and macrophages and the release of pro-inflammatory cytokines (e.g., TNF-α, IL-1β), ultimately leading to cell death (Ward et al., 2022). Ferritin (iron storage protein) and transferrin receptor (iron uptake protein) further influence neuronal iron homeostasis, which is often dysregulated in conditions such as AD and PD (Levi et al., 2024).

In addition, one of the mechanisms by which iron overload contributes to these disorders is epigenetic regulation, potentially presenting a therapeutic target and novel link between brain iron accumulation and the pathophysiology of both neurodegenerative and mental disorders. Epigenetics refers to the molecular modifications on DNA and chromatin that alter gene expression without changing the DNA sequence (Li, 2021). Epigenetic modifications can activate or silence gene expression, thereby altering cellular functions and disease progression in conditions such as cancer and neurological disorders (Lu et al., 2020; Grezenko et al., 2023). In the brain, excess ferrous iron can potentially disrupt the activity of both DNA methyltransferases (DNMT) and ten-eleven translocation proteins (TET) enzymes, impairing the DNA methylation and demethylation processes that regulate gene expression (Zhao et al., 2014). Iron overload may also deplete both GSH (reduced glutathione) and methyl donors, which are required for maintaining normal DNA methylation patterns (Aracena et al., 2006; Amanda, 2020). Therefore, it is hypothesized that brain iron overload is linked with epigenetic dysregulation (Ye et al., 2019), which could indirectly alter neurotransmission signaling pathways, thereby influencing mood and behavior.

As a result, maintaining brain iron homeostasis through iron chelation therapy has emerged as a promising strategy to attenuate oxidative stress, inhibit ferroptosis, and modulate disease progression, particularly in neurodegenerative disorders where iron accumulation is well documented (Zheng and Monnot, 2012; Zhang et al., 2021; Zeng et al., 2023). However, the role of iron in neuropsychiatric conditions remains less studied, as most clinical evidence suggests that iron deficiency rather than iron overload is associated with mood disorders (Duarte-Silva et al., 2025). Emerging studies suggest that instead of directly modulating neurotransmitter levels, brain iron overload could indirectly influence neurotransmission through epigenetic mechanisms (Ye et al., 2019; Wang Z. et al., 2023). Moreover, iron distribution in the brain is highly heterogeneous and region-specific; thus, targeted delivery approaches to specific brain region(s) or even cell type(s) could potentially modulate local iron levels without modifying iron homeostasis in the whole brain.

## 3 Iron chelation for brain iron accumulation

### 3.1 FDA-approved iron chelators

Current treatment of brain iron overload focuses on reducing iron levels and oxidative stress. This is primarily achieved through chelation therapy, which utilizes agents to bind and excrete iron, thereby preventing iron-induced organ damage. Several iron chelators are utilized clinically to ameliorate systemic iron overload disorders (such as hemochromatosis or transfusion iron overload) and have been considered or tested for addressing brain iron accumulation in neurodegenerative diseases. Three iron chelators are currently FDA-approved: deferoxamine (DFO), deferasirox (DFX), and deferiprone (DFP).

Deferoxamine, the first FDA-approved drug in 1968 (Parker et al., 2023), is administered via intravenous (IV) or intramuscular (IM) injection due to its poor oral bioavailability, which is one of the most significant limitations of the DFO treatment (Entezari et al., 2022). The strong iron-binding affinity of DFO (K_A_ = 4 × 10^16^) allows it to be the standard of care for acute iron toxicity for decades (Cheng et al., 2022). DFO is highly effective at mobilizing iron from the heart and liver (Maggio et al., 2011). However, it has a short plasma half-life of 20–30 min, necessitating continuous infusion over 8–12 h for 5–7 days a week, which significantly decreases patients’ compliance (Mobarra et al., 2016). Common side effects include local reactions at the injection site, hearing and vision impairment, and, less commonly, lung and kidney damage; therefore, regular monitoring is necessary, which imposes additional costs on patients (Koren et al., 1991; Bentur et al., 1991; Karnon et al., 2008). Deferasirox, approved in 2005, offers the possibility of once-a-day treatment as an oral chelator with a longer half-life. Although more convenient compared with DFO, DFX is significantly more expensive (Drugs.com, 2025). Due to its higher cost and being newer on the market, DFX may have less insurance coverage, making it less affordable for many patients (Tolley et al., 2010). Common adverse effects include skin rashes, gastrointestinal side effects, and an increase in serum creatinine, indicating the possibility of potential renal stress (Entezari et al., 2022). Deferiprone, as the first available oral iron chelator approved in Europe since 1999, had not been approved by the FDA until 2011 due to concerns about serious effects (Mobarra et al., 2016; Schafer, 2021). DFP is effective for long-term iron chelation therapy for transfusion iron overload in conditions like thalassemia and sickle cell disease (Crisponi et al., 2019). DFP is typically administered 3 times per day and is particularly effective in cardiac iron clearance (Pennell et al., 2006), potentially due to its low molecular weight and lipophilicity, which allow it to more easily penetrate myocardial cells and remove intracellular toxic labile iron stores (Pennell et al., 2011; Jamuar and Lai, 2012). Common side effects of DFP include gastrointestinal symptoms and agranulocytosis, and neutropenia (Ceci et al., 2019), requiring regular monitoring of blood counts. Table 1 summarizes the FDA-approved, clinically available iron chelators discussed above.

**TABLE 1:** Summary of FDA-approved iron chelators.

Chelators	Chemical structure	Half-life	Common side effects	FDA approval year
DFO	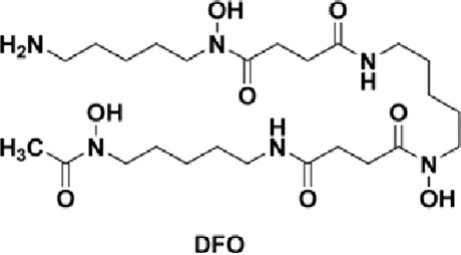	20–30 min (Abbina et al., 2019)	Local reaction, visual and hearing impairment	1968
DFX	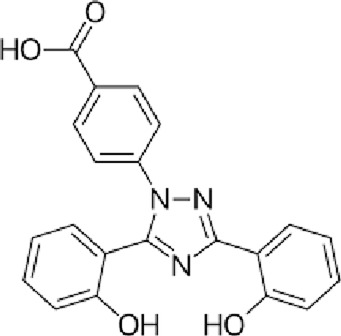	8–16 h (Piga et al., 2006)	Gastrointestinal issues, rashes, renal damage	2005
DFP	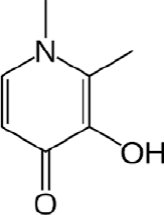	2–3 h (Poggiali et al., 2012)	Neutropenia, agranulocytosis, gastrointestinal discomfort	2011

### 3.2 Iron chelators for brain iron accumulation

In addition to systemic use, DFO and DFP have been investigated for neurological conditions associated with brain iron accumulation. DFO has demonstrated neuroprotective effects with the alleviation of behavioral deficits in a PD mouse model (Guo et al., 2016), although its clinical use for CNS disorders is limited by poor BBB permeability and oral bioavailability, as well as neurotoxicity at high doses (Freedman et al., 1988). DFP, as a more “conservative” iron chelator with milder iron binding affinity than DFO (Cabantchik et al., 2013), is smaller and considerably more lipophilic than DFO, which allows it to cross cell membranes and penetrate into subcellular organelles, making it one of a few iron chelators that can effectively cross the BBB and access CNS iron pools (Boyuklieva et al., 2024; Marchand et al., 2022). However, doses of DFP used in CNS conditions must be chosen carefully to avoid systemic toxicity.

Effective treatment schemes for neurological disorders using iron chelators are still lacking, primarily due to insufficient drug delivery across the BBB, which restricts most pharmaceutical agents from entering the CNS. Higher doses are often required to achieve higher CNS exposure, which in turn increases the risk of systemic toxicity. Moreover, chronic administration burdens (daily pills or injections for years) reduce compliance, especially in neurodegenerative conditions where cognitive impairment or motor deficits exist. The limited success of iron chelators used in neurological disorders has led to the exploration of optimized drug delivery systems that can overcome these barriers (Cecchelli et al., 2007). For example, clioquinol, a lipophilic iron chelator, is able to penetrate the BBB and reduce metal-induced oxidative stress and neuronal damage, but its use has been limited due to concerns about its serious neurotoxicity, including subacute myelo-optic neuropathy (SMON) (Cherny et al., 2001; Meade, 1975). These limitations have motivated researchers to investigate alternative delivery methods that can bypass the BBB while ensuring sufficient patient compliance, preferably avoiding invasive routes like injections.

## 4 Iron chelation therapies for CNS disorders via different routes

Delivering therapeutic agents to the CNS requires the delivery system to be capable of penetrating the brain to reach target disease sites, facing the challenge of two barriers between blood and the brain–the BBB and the blood-cerebrospinal fluid (CSF) barrier. The BBB, composed of tightly joined endothelial cells, astrocytic end-feet, and pericytes, serves as a selective barrier that regulates the passage of substances from the bloodstream into the brain parenchyma (Abbott et al., 2010). Small molecules such as carbohydrates, amino acids, and hormones pass across the BBB using endothelial carrier-mediated transporters (Oldendorf, 1971), while macromolecules such as transferrin and insulin use endothelial receptor-mediated transport (Banks, 2012). Furthermore, ion concentrations in the CNS are controlled by endothelial ion transporters and channels (Nance et al., 2022). Similarly, the blood-CSF barrier is formed by the choroid plexus epithelium, controlling the exchange between the blood and the CSF, although allowing more molecules to cross compared to the BBB (Pardridge, 2011).

**FIGURE 1:**
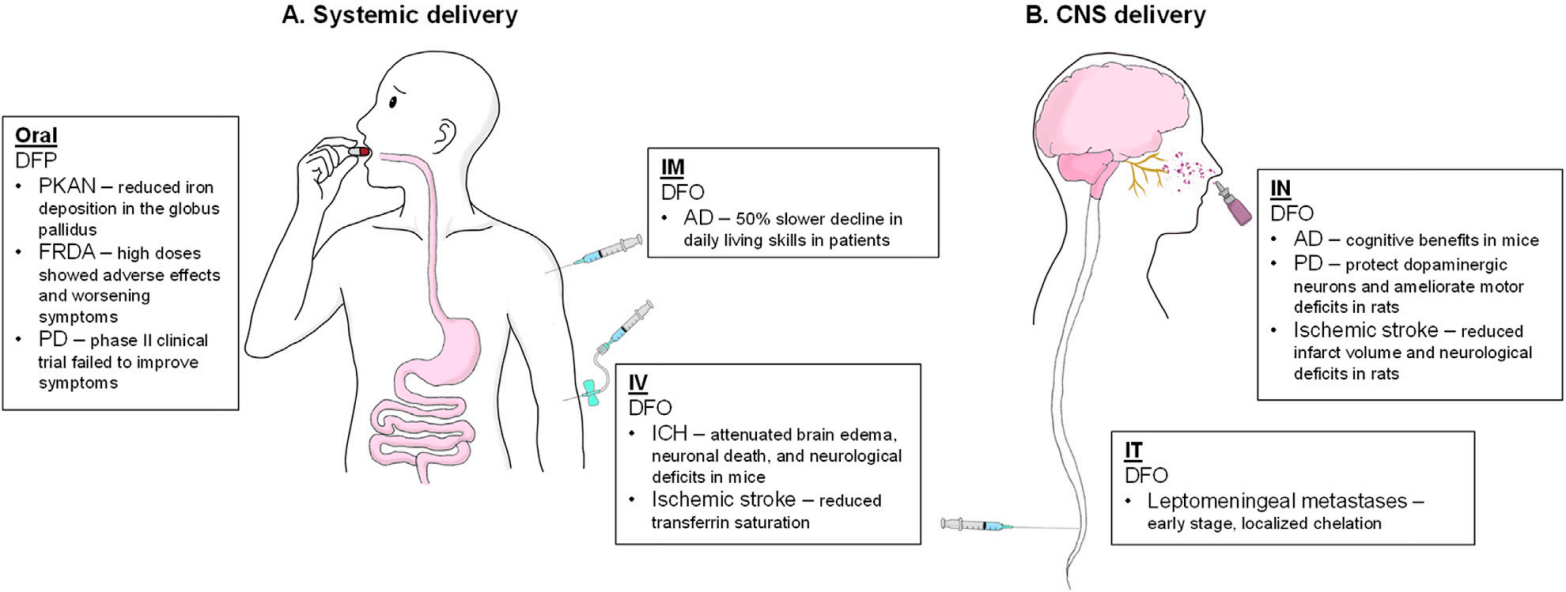
Iron chelators in the treatment of CNS disorders via different administration routes. **(A)** Systemic (indirect) delivery routes; **(B)** CNS (direct) delivery routes. Abbreviations: AD, Alzheimer’s disease; DFP, deferiprone; DFO, deferoxamine; FRDA, Friedreich’s ataxia; ICH, intracerebral hemorrhage; IM, intramuscular; IN, intranasal; IT, intrathecal; IV, intravenous; PD, Parkinson’s disease; PKAN, pantothenate kinase-associated neurodegeneration.

Once successfully passing through these barriers and entering the brain, therapeutic agents encounter additional challenges within the brain’s interstitial environment. The transition from the lipid environment of the BBB endothelial cell membrane to the aqueous interstitial fluid of the brain’s extracellular space necessitates further consideration of a drug and its delivery vehicle’s physicochemical properties, including size, surface charge, shape, and molecular weight (Sykova and Nicholson, 2008; Patel et al., 2012). Moreover, factors such as fluid dynamics, pH, and disease-induced pathological alterations in the brain’s microenvironment (i.e., changes in enzymatic activity, impairment in vascular function, and inflammation) can significantly influence drug distribution and efficacy (Nance et al., 2022). Methods to deliver iron chelators to the CNS include direct (such as intranasal and intrathecal) and indirect (such as oral and injections) routes (Figure 1) (Rohani et al., 2017; Pandolfo et al., 2014; Millan et al., 2021; Li et al., 2022; Hanson et al., 2009; Guo et al., 2013a; Fine et al., 2024; Febbraro et al., 2013; Devos et al., 2022; Crapper McLachlan et al., 1991; Clinicaltrials.gov, 2025).

### 4.1 Oral administration

Oral delivery is the most convenient route for chronic therapy, and several iron chelators are orally available for both clinical and preclinical studies. Orally administered chelators undergo gastrointestinal absorption and systemic distribution, with their ability to reach the CNS depending on their physicochemical properties and physiological factors.

Oral DFP has shown the most promising results for chelating brain iron due to its good absorption and distribution in the brain. In a pilot trial, DFP orally administered 15 mg/kg twice daily showed reduced iron deposition in the globus pallidus in patients with pantothenate kinase-associated neurodegeneration (PKAN), a rare disease characterized by neurodegeneration with brain iron accumulation (NBIA) (Abbruzzese et al., 2011). Interestingly, systemically delivered DFP exhibited acute anti-depressant activity in a mouse model of depression without modifying brain and blood iron levels, suggesting an iron-independent mechanism (Uzungil et al., 2022). These results indicate that DFP can interact with brain iron and potentially ameliorate neurodegenerative and behavioral alterations.

However, DFP demonstrated inconsistent efficacy in other neurodegenerative diseases (Boddaert et al., 2007). For instance, in a six-month randomized controlled trial of 72 patients with Friedreich’s ataxia (a neurodegenerative disorder characterized by mitochondrial iron accumulation), 20 mg/kg/day DFP was well tolerated but higher doses (40 and 60 mg/kg/day) were associated with increased adverse events and worsening of ataxia symptoms (Pandolfo et al., 2014). A recent phase II clinical trial in Parkinson’s disease, which assessed DFP in 372 newly diagnosed, untreated PD patients over 36 weeks, revealed that DFP not only failed to improve disease symptoms but was associated with a worsening of motor and non-motor symptoms compared to placebo (Devos et al., 2022). While DFP effectively reduced iron levels in the substantia nigra (Negida et al., 2024), this did not translate into clinical benefit in PD patients. A proposed explanation is that iron is essential for the activity of tyrosine hydroxylase, the enzyme responsible for dopamine synthesis; thus, chelation of iron by DFP may have indirectly impaired dopamine production, exacerbating PD symptoms (Galasko and Simuni, 2022). This hypothesis is supported by observed increases in prolactin levels among DFP-treated patients, suggesting a reduced dopaminergic activity (Devos et al., 2022).

DFX is also orally available and highly protein-bound (Gottwald et al., 2020), which potentially limits the efficacy of BBB penetration. A clinical trial in aceruloplasminemia (both liver and brain iron overload) showed DFX effectively normalized hepatic iron but had no effect on brain iron levels, indicating the limited CNS distribution of oral DFX (Finkenstedt et al., 2010). In preclinical Alzheimer’s models, chronic oral DFX showed minimal benefit on cognitive or pathological outcomes, with only a modest trend toward reduced tau phosphorylation and no improvement in memory function (Kwan et al., 2022). DFO, on the other hand, has a higher molecular weight compared to other chelators and is highly hydrophilic; thus, it has poor absorption via the GI tract and is not suitable for oral administration. Nonetheless, a mouse study where low-dose oral DFO was added to chow for 2 weeks showed decreased brain iron by ∼18% by MRI and decreased multiple major iron metabolism-related protein levels (Thorwald et al., 2025). Clinically, DFO is not given by the oral route due to its extremely poor absorption.

The long-term safety issues associated with the chronic administration of oral chelators require consideration. While oral DFP has a risk of hematological toxicity (Ruivard and Lobbes, 2023), oral DFX can cause hepatic and renal toxicity and gastrointestinal bleeding with chronic use (Yusuf et al., 2023). In addition, DFX does not enter the brain, making it unsuitable for neurodegenerative applications. Furthermore, a key limitation of oral administration is the off-target effect, where most of the drugs may act on peripheral organs before they can reach the CNS. Especially for individuals with high peripheral iron levels, DFP might be less efficacious due to the potential iron chelation in peripheral tissues.

In summary, oral administration of iron chelators (especially DFP) provides a non-invasive and promising dosing route for treating brain iron accumulation, but it requires prolonged treatment to achieve effective concentrations, close safety monitoring due to chronic treatment at relatively large dose, and its efficacy might be constrained by peripheral iron binding and the need for sufficient BBB penetration.

### 4.2 Intravenous administration

Intravenous (IV) administration ensures 100% bioavailability of the drug in the bloodstream and allows for the most rapid and highest plasma concentrations. However, entry into the brain still depends on the permeability of the BBB. Large hydrophilic chelators, such as DFO (a highly polar molecule with a molecular weight of ∼560 Da), exhibit poor BBB permeability when administered intravenously, remaining in the circulation and extracellular space, while only minimal amounts can cross into the brain parenchyma. Apart from DFO, other iron chelators (DFP and DFX), which are orally available, are rarely given by IV administration.

In certain circumstances (such as hemorrhage and trauma), the BBB can be impaired, thus allowing greater penetration of IV DFO into the brain (Li et al., 2022). In animal studies of intracerebral hemorrhage (ICH), DFO attenuates brain edema, neuronal death, and neurological deficits (Li et al., 2022; Yu et al., 2015). In clinical trials in patients with ICH, DFO is also found to be highly tolerated and has a higher chance of a positive clinical outcome after consecutive days of IV infusion, although further confirmation is needed for its efficacy (Selim et al., 2011; Selim et al., 2019; Yu et al., 2017; Shirani et al., 2025). While generally considered safe, long-term or high-dose DFO administration can still lead to neurotoxicity or organ damage (Zhang et al., 2025), and it requires slow infusion due to the risk of hypotension (Gerhardsson, 2022). Moreover, because continuous or frequent infusion is needed due to DFO’s short half-life, outpatient use is impractical except via special devices.

In summary, while systemic IV DFO is invasive and non-specific for brain delivery for chronic brain iron overload, it may have potential applications for immediate effect and is useful in acute neurological damage where iron is rapidly released and accessible (e.g., acute iron poisoning or ICH).

### 4.3 Intramuscular administration

Intramuscular (IM) injection is an alternative parenteral route that deposits the drug into muscle tissue for slow absorption into the circulation. IM administration bypasses first-pass metabolism and can provide a prolonged release into the bloodstream compared to IV bolus injection. However, the IM route still faces the challenge of BBB penetration and relies on the transport to the CNS after systemic absorption. Like the IV route, no CNS-oriented IM administration is reported for either DFP or DFX, as oral administration is available and patient-friendly for these chelators. Thus, IM delivery is mainly used for DFO when IV access is unavailable or to avoid IV-related risks (e.g., frequent doses due to short half-life).

A 2-year clinical study in 1991 used low-dose IM DFO (125 mg b.i.d., 5 days/week) in AD patients, which showed a significantly slower (∼50%) decline in daily living skills, suggesting a disease-slowing effect (Crapper McLachlan et al., 1991). This was the first clinical study, hinting that brain iron chelation might modify disease progression in AD, although no follow-up clinical studies have been conducted over the past 30 years, potentially due to the constraints of time and funding over the 2-year treatment period (Percy et al., 2011). Apart from this successful clinical trial, few modern trials use the IM route for iron chelation in brain disorders, likely due to the availability of IV infusion pumps and controlled-release devices that slow down the rapid elimination profile of DFO.

Repeated IM injections are painful and inconvenient, and other local side effects such as tissue irritation or fibrosis at injection sites are also concerns, leading to low long-term patient compliance (Farr and Xiong, 2021). Overall, IM delivery has the potential to achieve clinical effects but does not provide an advantage over other routes in targeting brain iron.

### 4.4 Intrathecal administration

Intrathecal (IT) delivery involves administering the chelator directly into the CSF (often via lumbar puncture or an implanted reservoir), thereby bypassing the BBB (Fowler et al., 2020). By this direct route, very high concentrations of the drug can be achieved in the CSF and adjacent brain tissue, especially around the ventricles and spinal canal. Intrathecally administered iron chelators (such as DFO) are expected to extensively chelate iron in the CSF and outer brain regions (such as cortex) and may eventually reach deeper brain regions via CSF circulation.

Molecules administered by intrathecal administration still encounter barriers within the subarachnoid space for drug penetration from CSF into brain parenchyma, but IT dosing creates a favorable concentration gradient for drugs to diffuse. There is limited clinical data published on intrathecal iron chelation in neurodegeneration, and no iron chelator is officially approved for IT use, given that this is a rather invasive experimental approach. In an ongoing phase 1a/1b trial of leptomeningeal metastases (malignant cells in the CSF), IT DFO will be tested through an Ommaya reservoir to determine the highest dose of DFO that can be tolerated (Clinicaltrials.gov, 2025), but full results are still pending. In routine clinical practice, IT administration is more commonly used for analgesics or antibiotics (Fowler et al., 2020). Because of the invasive nature of IT administration, regulatory approval for clinical trials would require clear evidence of benefit and safety, especially when less invasive approaches (oral/intranasal) are available.

Delivering drugs directly into the CSF also raises significant safety concerns. IT DFO could potentially cause neurotoxicity if the local concentration is too high–high doses of systemic DFO have been associated with neurotoxic effects (e.g., hearing loss, seizures) (Zhang et al., 2025), and these risks would potentially be higher through direct CNS exposure. Therefore, although very potent in terms of directly tackling brain iron overload, a non-invasive but direct method is necessary for CNS delivery.

### 4.5 Intranasal administration

Drugs delivered to the olfactory region via IN can reach the olfactory bulb and other CNS regions within minutes via intracellular and extracellular pathways along the olfactory nerves or diffusion (especially for lipophilic small molecules), allowing for a higher concentration of the therapeutic agents in the brain compared to the bloodstream. DFO has been the most extensively studied in IN delivery experiments. IN DFO in rats resulted in drug concentration in the cortex approximately 10-fold higher than an equivalent intravenous dose, with lower systemic concentrations and systemic side effects (Hanson et al., 2009).

A portion of intranasally delivered DFO travels directly into the brain, whereas the rest is systemically absorbed via the nasal mucosa. After 25 min of intranasal delivery, relatively high levels of DFO were found in the frontal cortex (2.1–4.7 µM) and hippocampus (1 µM), higher than those in peripheral areas (kidney 0.63 µM, liver 0.37 µM) (Hanson et al., 2009). Additionally, as a hydrophilic molecule, DFO has a poor cell permeability and tends to remain in extracellular fluid, which may still limit its efficacy (Hanson et al., 2009; Liu et al., 2018).

Studies on IN delivery of DFO have shown promising results in mitigating cognitive deficits and motor impairments in neurodegenerative diseases such as AD (Guo et al., 2013a; Hanson et al., 2012; Fine et al., 2012), PD (Fine et al., 2014), and intracerebral hemorrhage (ICH) (Hanson et al., 2009). For example, intranasal DFO treatment in mouse models of AD improved memory performance in spatial mazes and reduced pathological hallmarks, including brain Aβ levels and tau hyperphosphorylation (Fine et al., 2024; Fine et al., 2012; Fine et al., 2015; Fine et al., 2017; Guo et al., 2013b). DFO did not dramatically decrease total brain iron content, but it appeared to redistribute iron from vulnerable regions of disease pathology (Guo et al., 2015), suggesting that DFO can possibly stabilize iron in forms that are less neurotoxic. In rat models of PD, IN DFO has been reported to protect dopaminergic neurons in the substantia nigra and ameliorate motor deficits, suggesting IN DFO reaches the midbrain regions at pharmacologically active concentrations (Febbraro et al., 2013). Apart from neurodegeneration, IN DFO has also been tested in acute CNS injury models. In a rat model of ischemic stroke after middle cerebral artery occlusion (MCAO), IN DFO significantly reduced infarct volume and neurological deficits (Hanson et al., 2009).

Apart from IN DFO, recent studies have explored the possibilities of other chelators administered via the nasal route. For example, Boyuklieva et al. developed biodegradable nanocomposite microspheres encapsulating DFP to enhance mucoadhesion and sustain drug release for nasal administration (Boyuklieva et al., 2024), although *in vitro* and *in vivo* efficacy data are still lacking. Meanwhile, an early-phase human case trial by Amoushahi et al. also reported brain de-ironization efficacy of the nasal spray of DFP in a patient with multiple system atrophy (Amoushahi et al., 2024).

Intranasal delivery of iron chelators is promising, supported by their use for decades in other systemic contexts. A growing body of translational research suggests that IN delivery can achieve CNS therapeutic effects while minimizing systemic exposure, allowing patients to experience fewer adverse effects, such as anemia or gastrointestinal upset, which are common concerns with oral or intravenous chelators. As of now, only small-scale case studies have been conducted for IN administration of iron chelators, but more progress in clinical translation is anticipated. Overall, IN DFO is expected to have a better safety profile than systemic high-dose DFO. IN DFP provides another promising potential because of its higher lipophilicity and smaller molecular weight. Patients will still need to be monitored for both local effects (mucosal changes or olfactory disturbances) and any systemic absorption effects (e.g., serum iron and blood counts) when intranasal chelation is tested in clinical studies. More details about intranasal delivery, including its molecular mechanism, advantages, and limitations, are discussed in the following section.

## 5 Pharmacology of intranasal chelators

The route of administration significantly affects the pharmacokinetics, efficacy, and toxicity of iron chelator therapy for CNS disorders. Oral administration has the benefit of high patient compliance and has demonstrated the potential to target brain iron pathology, especially with BBB-permeant agents like DFP. However, peripheral distribution decreases the efficacy of oral therapy, requiring high doses, which causes potential systemic side effects (e.g., neutropenia). Intravenous and intramuscular routes deliver chelators into the circulation more directly, which is useful for systemic iron removal but still only offers limited brain uptake in most chronic conditions. Repeated IV/IM doses indeed suggested efficacy for DFO in Alzheimer’s disease, but such invasive regimens are not practical long-term and carry significant risks. Intrathecal administration, while conceptually the most direct way to approach brain iron, is highly invasive and currently studied only in experimental scenarios–it may be considered in extreme cases or acute CNS iron toxicosis, but it is not a routine approach due to the safety and logistical challenges.

Intranasal delivery provides an innovative strategy that bridges the gap between efficacy and safety. By reaching the BBB via olfactory pathways, IN chelators like DFO achieve CNS exposure faster and better than systemic routes, yet without the need for surgery or injections. This route is a promising alternative for treating neurodegenerative and neurovascular conditions characterized by iron dysregulation. Nonetheless, even though an IN chelator enters the brain, its distribution is unlikely to be region-selective, which is a major limitation. In addition, since iron is heterogeneously distributed across brain regions and cell types (Levi et al., 2024), potential off-target chelation could result in iron depletion in brain regions where iron supports physiological function and consequently affect neuronal and glial metabolism, neurotransmitter synthesis, or cell signaling. Thus, while IN chelation is promising for directly targeting the brain and CNS, it still requires careful consideration of formulation strategies (e.g., nanoparticle targeting or controlled-release systems) to improve site specificity, especially for drugs with a narrow therapeutic window. Moreover, given that drug exposure is expected to be highest in the olfactory bulbs compared to other brain regions, IN chelator therapy may be particularly beneficial for disorders involving early olfactory bulb pathology or localized iron dysregulation near the nasal region (Deng et al., 2024).

### 5.1 Mechanism of intranasal delivery

Since William Frey II proposed intranasal administration in 1991 (Frey, 1991), this technique has emerged as a novel, non-invasive delivery system, bypassing the BBB and the systemic first-pass effect, allowing rapid onset of action. The nasal cavities of the nose are anatomically divided into three regions: 1) Vestibular region: located just inside the nostrils, has a limited surface area resulting in filtration of inhaled particles and minimal drug absorption; 2) Respiratory region: the majority of nasal cavities that has a large surface area and rich vascularization, making it the primary site for systemic drug absorption; 3) Olfactory region: a small region located on the superior aspect of the nasal cavities and is crucial for direct nose-to-brain delivery. While this is only 10% of the area in humans, the olfactory region can be up to 50% of the total area in rodents, making them a suitable model for intranasal administration research (Chamanza and Wright, 2015). This region contains olfactory neurons that extend cilia into the mucus layer and project axons through the cribriform plate to synapse in the olfactory bulb, allowing drugs to bypass the BBB and reach the CNS directly via the olfactory and trigeminal nerve pathways (Crowe et al., 2018).

**FIGURE 2:**
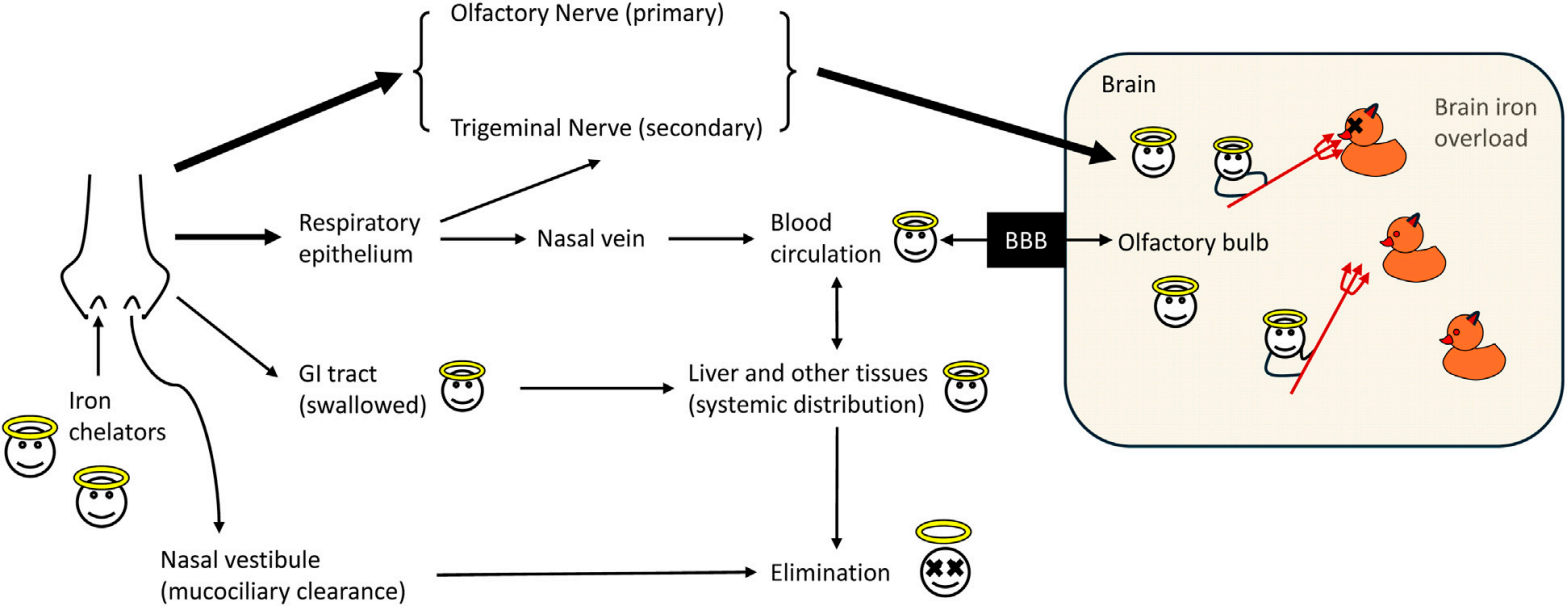
Pathways of iron chelators after intranasal administration. Once administered intranasally, the chelators reach the nasal mucosa where a portion may be excreted out by mucociliary clearance through the nasal vestibule. The remaining chelators then permeate the upper region of the nasal mucosa to reach the nasal epithelium. From there, they directly enter the CNS via two pathways: 1) the olfactory nerve to the olfactory bulb, and 2) trigeminal nerve to the brain stem. The highly vascularized nature of the nasal cavity also allows a minor fraction of drugs to be absorbed into systemic circulation through the nasal veins. Among those, lipophilic small molecule drugs can potentially pass through the hydrophobic blood-brain barrier (BBB) and tight junctions and eventually access the CNS. Excess chelators delivered to the nasal cavity may be swallowed and subsequently absorbed by the gastrointestinal (GI) tract, followed by distribution to the liver and other tissues and elimination.

The transport of drug molecules from the nasal cavity to the brain involves two primary pathways: intracellular and extracellular. In the intracellular pathway, drugs are internalized by olfactory neurons through endocytosis, transported to the end of the axon, and subsequently released into the brain via exocytosis (Crowe et al., 2018). Simultaneously, in the extracellular pathway, drugs penetrate the nasal epithelium and enter the lamina propria, then move along the perineural spaces surrounding olfactory and trigeminal axons, allowing both small and large molecules to reach the CNS. This dual mechanism allows for a more rapid and efficient delivery route of pharmaceutical agents to enter the brain and exert therapeutic effects (Figure 2).

### 5.2 Clinical application of intranasally delivered drugs

Although IN delivery of iron chelators remains in the early preclinical stages and has not yet entered clinical trials, the broader field of IN route has gained increasing attention over the past decade, as evidenced by a notable rise in related clinical studies. The number of clinical trials in IN administration between 2015 and 2025 has nearly doubled compared to the 2005–2015 period, increasing approximately from 520 to 910 trials. This increase includes patients of various age and sex groups, notably among both children and adults.

The global market for nasal drug delivery in patients was valued at approximately $85.89 billion in 2025 and is projected to reach $119.56 billion by 2029 at a compound annual growth rate (CAGR) of 8.6% (The Business Research Company, 2025). The U.S. Food and Drug Administration (FDA) has approved several intranasal drugs for acute conditions, including naloxone for opioid overdose and fentanyl for pain management (Madden et al., 2023; Crellin et al., 2010). A comprehensive list of FDA-approved IN drugs is provided in Table 2.

**TABLE 2:** FDA-approved intranasal (IN) drugs.

Therapeutic area	Drug name	Application	FDA approval year
CNS			
Opioid overdose	Naloxone	Reduce drug overdose death	2015, OTC since 2023
	Nalmefene hydrochloride	Reverse opioid overdose (opioid receptor antagonist)	2023
Depression	Spravato	Antidepressant for treatment-resistant depression	2019
Seizure	Nayzilam	Acute treatment of seizure clusters	2019
	Valtoco	Short-term treatment of epilepsy cluster seizures	2020
Pain management	IMITREX	Acute treatment of migraine	1997
	ZOMIG	Acute treatment of migraine in pediatric patients	2015
	Trudhesa	Acute treatment of migraine	2021
	Zavzpret	Acute treatment of migraine	2023
	Sprix	Acute moderate to severe pain	2010
	Lazanda	Breakthrough pain in cancer patients	2011
Others	Nicotine Spray	Smoking cessation aid	1996
Peripheral			
Allergy	QNASL	Seasonal allergic rhinitis (SAR) and perennial allergic rhinitis (PAR)	2012
	Flonase	Corticosteroid for nasal symptoms	1994, OTC since 2014
	Astepro	Antihistamine for nasal symptoms of allergies	2021
	Neffy	Emergency treatment of allergic reactions (type I) including life-threatening anaphylaxis	2024
Others	FluMist	Influenza vaccine	2003
	Baqsimi	Emergency treatment of severe hypoglycemia in diabetes	2019

The recent application of FDA-approved intranasal drugs still indicates a trend toward treatments for acute conditions requiring rapid onset of action. However, studies into their potential for treating various other CNS-related diseases are actively ongoing. Notably, the intranasal delivery of drugs, including small molecules, nanoformulations and gene delivery agents, has shown promising outcomes in various rodent models of several neurological pathologies. For instance, preliminary studies suggest the feasibility of IN insulin to improve age-related cognition and memory deficits (Maimaiti et al., 2016; Reger et al., 2006; Craft et al., 2017; Keller et al., 2022), highlighting an opportunity to optimize intranasal formulations to effectively target the CNS. Intranasal delivery of cationic nanoemulsion-encapsulated therapeutics has shown anti-neuroinflammatory effects in the CNS in rodents (Yadav et al., 2016; Yadav et al., 2019). Such advancements have led to our interest in using this technique to manage brain iron levels in neurological diseases, addressing a critical gap in current treatment strategies.

### 5.3 Advantages of intranasal delivery

Intranasal (IN) delivery offers several advantages over other common routes, such as oral administration, particularly in enhancing therapeutic efficacy and patient compliance. One significant benefit is the rapid onset of action facilitated by the nasal cavity’s rich vascularization and large surface area in the submucosa, which allows rapid drug absorption. Molecules administered by IN can reach the CSF and brain parenchyma within minutes of dosing, offering a rapid therapeutic onset, which might be beneficial in acute settings, including acute brain injuries or stroke. This characteristic is especially beneficial in emergency situations or for conditions requiring immediate relief, such as acute pain or migraines. Additionally, IN delivery bypasses the gastrointestinal tract and hepatic first-pass metabolism, potentially leading to improved bioavailability of oral iron chelators like DFP (Pires et al., 2009; Grassin-Delyle et al., 2012). This means that lower doses may achieve the desired therapeutic effect, thereby reducing systemic side effects and enhancing treatment efficacy (Agrawal et al., 2018).

Furthermore, the non-invasive nature of IN administration enhances patient comfort and adherence to treatment regimens that provide direct access to the CNS. Unlike orally delivered chelators, which must survive harsh GI conditions and extensive hepatic first-pass effect before reaching systemic circulation, IN drugs only need to cross the nasal epithelium or can directly access the brain through neuronal pathways. This route offers more flexibility in the physicochemical properties of drug formulations. For chelators, such as DFO, which has a short plasma half-life and therefore requires frequent doses, the IN route provides a safe and efficacious delivery method with higher patient compliance.

Systemically delivered drugs must cross the BBB, favoring small (under 500 Da) and lipophilic molecules for optimal absorption (Alqahtani et al., 2021), whereas IN delivery allows both small and large molecules, including peptides and proteins, to bypass the tightly sealed endothelial barriers and potentially reach the CNS directly. Pharmacokinetics studies showed that IN delivery of Cyclosporine-A nanoemulsion has a significantly higher brain-to-blood concentration ratio and lower systemic tissue distribution compared with IV injection route (Yadav et al., 2015). However, drugs with lower molecular weights and higher lipophilicity are still favored to enhance the permeability through the nasal mucosa. Moreover, while oral drugs need to be stable enough to survive the enzymatic degradation in the stomach and liver, IN-delivered drugs face fewer challenges due to the relatively lower expression of enzymes in the nasal mucosa.

In summary, while both oral and IN delivery require careful yet distinct considerations of formulation, IN delivery provides a viable alternative for drugs, especially those targeting the CNS. More detailed information on the requirements for oral and intranasal formulations is summarized in Table 3.

**TABLE 3:** Considerations of oral and intranasal formulations.

Key characteristics	Oral	Intranasal
Barriers	Stomach acid, digestive enzymes, liver metabolism, BBB	Nasal epithelium, mucociliary clearance
Formulation flexibility	Strict (Lipinski’s rule of five)	Relaxed size and lipophilicity requirements
Type of drugs	Small, lipophilic	Small to large molecules
Onset of action	Slow (30 min to hours)	Fast (minutes)
Dose precision	High (controlled tablets, capsules)	Lower (affected by mucociliary clearance and nasal variability)
Formulation considerations	Protect from acid and enzymes, improve solubility	Extend residence time, enhance permeability
Typical problems	Low bioavailability, degradation	Fast clearance, dose variability

### 5.4 Considerations for intranasal delivery

IN delivery provides a non-invasive and accessible dosing route for medication administration, but it also has several concerns that necessitate careful consideration. One primary concern is the limited volume capacity of the nasal cavity, typically allowing less than 1 mL per nostril for human patients and less than 10 µL for mice (Tsze et al., 2017; Miller et al., 2012). This limits the dose of drug that can be delivered at one time, requiring that formulations appropriate for intranasal delivery be highly concentrated, soluble and stable to ensure effective dosing within the limited volume. Additionally, the nasal mucosa’s rapid mucociliary clearance (10–15 min) limits the residence time of the drugs and absorption efficiency (Riese et al., 2014). Formulation strategies, including the incorporation of mucoadhesive agents (such as chitosan or carbopol) or liposomes, are often applied to enhance drug absorption (Boddupalli et al., 2010; Taha et al., 2023).

The physicochemical properties of drugs also influence their suitability for IN delivery. The limited absorption of the nasal cavity restricts the amount of drug being effectively absorbed, making class I drugs (high permeability, high solubility), according to the Biopharmaceutics Drug Disposition Classification System (BDDCS), particularly suitable for nasal delivery (Bitter et al., 2011). However, the requirement for lipophilicity is less strict compared to systemic delivery due to the direct nerve routes available via IN administration. Special formulation strategies are often employed to enhance absorption. For example, hydrophilic drugs (like DFO) might be incorporated with absorption enhancers such as surfactants or mucoadhesive polymers to enhance permeability through the mucosa; otherwise, the water-soluble compound might drip out of the dose. Lipophilic drugs are often encapsulated in carriers like liposomes, nanoparticles, or emulsions to improve solubility and retention time and reduce the mucociliary clearance (Ugwoke et al., 2001). Nanoparticles can also protect the drug from enzymatic degradation in the nasal mucosa and facilitate transport across cellular barriers (Boyuklieva et al., 2024). However, while smaller particle size is more optimal for both olfactory bulb axonal transport and mucosal layer penetration (Mistry et al., 2009; D'Souza et al., 2021), very small particles (<200 nm) might be inhaled into the lungs or cleared too rapidly from the nose, while large microparticles (5–10 µm) have better nasal retention but may not be able to transverse into the brain tissue (Frank et al., 2012).

The hydrophilicity of DFO also limits its permeability through the cells, including neurons (Kontoghiorghes and Kontoghiorghe, 2016), requiring higher doses that can increase the risk of severe systemic side effects such as cardiomyopathy, hepatic or renal failure, and neurotoxicity (Cheng et al., 2022; Bentur et al., 1990). While IN delivery of iron chelators does target the brain, it may achieve higher drug concentrations in regions near the entry points (e.g., the olfactory bulbs and frontal cortex) and potentially lower concentrations in distant brain areas (Xu et al., 2024). CNS disorders with widespread iron accumulation in areas, including the basal ganglia, might not fully benefit if the drug mainly targets certain areas. Therefore, IN chelators could be more effective for neurological symptoms associated with problems in the olfactory bulb, prefrontal cortex, or hippocampus (cognitive impairment and psychiatric symptoms) (Bhattarai et al., 2022; Salimi et al., 2019; Hasegawa et al., 2022) than for ones like Parkinson’s disease, where the main pathology is in the midbrain. More studies need to be conducted to test whether repeated dosing could help the drug penetrate further over time.

Although IN delivery of DFO appears to be well tolerated in animal models, repeated administration can potentially cause nasal irritation or epistaxis (nosebleed), which can induce inflammation or reduced drug uptake. However, iron chelators are relatively small molecules compared with proteins, and no significant neurotoxicity or immunogenicity due to IN DFO has been reported. The potential irritation can be minimized by adjusting the pH of the nasal formulation to 4.5–6.5 to match the normal pH of the nasal mucosa (Vidyavathi et al., 2021). Furthermore, the tonicity and viscosity of the formulation are critical factors that affect drug absorption and patient comfort (Dhuria et al., 2010).

Moreover, the dose from intranasal delivery is relatively hard to control compared to oral delivery. Variability in mucociliary clearance speed and mucus thickness, often affected by patient conditions such as anatomical differences, inflammation or allergies, and even the technique of administration (angle of spray, depth of insertion) (Riese et al., 2014), can alter drug absorption and uptake into the brain. Inconsistent delivery could lead to variable therapeutic outcomes. Thus, the development of suitable delivery devices is needed to target the appropriate region of the nasal cavity effectively (Scheibe et al., 2008).

Despite these challenges, IN delivery is predominantly used for emergency medicine in clinical settings due to its rapid onset, ease of administration, and ability to bypass hepatic first-pass metabolism. These advantages have made IN delivery ideal for urgent treatments like sedation, analgesia, opioid overdose, seizures and breakthrough pain relief in cancer patients (Doe-Simkins et al., 2009; Pavis et al., 2002), yet still providing a compelling rationale for further development of IN iron chelation therapies for CNS disorders.

### 5.5 Conclusion and future direction

Recognizing the limitations of conventional delivery routes in targeting the CNS, IN administration provides a non-invasive alternative that not only bypasses the BBB but also avoids hepatic first-pass metabolism. This route offers a more rapid and efficient pathway, potentially increasing the CNS bioavailability of drugs for neurological diseases where therapeutic windows are narrow and rapid intervention may be critical.

While IN delivery has been explored in several CNS drugs (Jones et al., 2017; Ortega et al., 2021), its application in iron chelators offers unique advantages, particularly for treating brain iron overload associated with psychiatric and neurodegenerative conditions. IN administration may maximize brain-specific absorption while minimizing systemic exposure, ensuring the effectiveness and safety of the therapy. This approach improves the delivery of therapeutics into CNS, as well as offers alternative approaches for the treatment of various neurodegenerative conditions.

Beyond FDA-approved drugs, several experimental iron chelators have demonstrated strong potential for intranasal brain targeting. ATH434 (formerly PBT434), which recently completed a Phase 2 clinical trial for multiple system atrophy (MSA), acts as an orally available iron chaperone that redistributes labile iron in preclinical models of MSA and PD (Bailey et al., 2021; Pall et al., 2024; Beauchamp et al., 2022). Its oral bioavailability and moderate BBB penetration suggest IN delivery could further improve its CNS distribution. VK-28, another brain-permeable iron chelator, has been shown to protect dopaminergic neurons and reduce iron-induced lipid peroxidation in 6-OHDA PD models, indicating its suitability for nasal formulations for enhanced neuronal targeting (Li et al., 2017; Shachar et al., 2004). N,N’bis-(2-mercaptoethyl) isophthalamide (NBMI), a lipophilic thiol-based chelator with selective affinity for ferrous iron, has demonstrated superior brain chelation efficacy in a mouse model of brain iron overload, making it a promising candidate for IN formulations if its solubility limitations can be addressed (Cheng et al., 2022). Taken together, these compounds show promising potential for nasal formulations, with the potential to enhance brain targeting and accelerate their clinical translation for iron-related neurological disorders.

The translation from animal studies to human therapy may face some hurdles, as rodents have a significantly larger olfactory epithelial surface area relative to their nasal cavities compared to humans. This anatomical difference may render intranasal delivery less efficient in humans. Future research will need to confirm that IN chelation can remove or redistribute iron in the human brain and can be translated into clinical improvements (slower neurodegeneration or better neurological condition). Additionally, the application of IN delivery for other metal-related neurotoxicity, such as copper overload in Wilson’s Disease, could also be explored. Careful optimization of IN dosing regimens will be essential for maintaining efficacy, minimizing side effects, and increasing patient compliance. The nasal route avoids systemic side effects, but it may introduce local side effects. Long-term safety will also be critical if IN chelation therapy is to be considered for chronic use.

Overall, IN delivery presents a promising strategy to expand therapeutic options for CNS disorders with complex pathological factors such as metal dysregulation. Ongoing research is actively addressing key challenges, including formulation optimization, long-term safety, and consistent delivery in humans. If successful, IN delivery has the potential to become a transformative strategy in the treatment of CNS disorders.
